# Inoculation of pear flowers with *Metschnikowia reukaufii* and *Acinetobacter nectaris* enhances attraction of honeybees and hoverflies, but does not increase fruit and seed set

**DOI:** 10.1371/journal.pone.0250203

**Published:** 2021-04-22

**Authors:** Agneta Colda, Sofie Bossaert, Christel Verreth, Bart Vanhoutte, Olivier Honnay, Wannes Keulemans, Bart Lievens

**Affiliations:** 1 Division of Crop Biotechnics, Laboratory for Fruit Breeding and Biotechnology, Department of Biosystems, KU Leuven, Leuven, Belgium; 2 Laboratory for Process Microbial Ecology and Bioinspirational Management, Department of Microbial and Molecular Systems, Center of Microbial and Plant Genetics, KU Leuven, Leuven, Belgium; 3 Research Center for Fruit Growing, Sint-Truiden, Belgium; 4 Division of Ecology, Evolution and Biodiversity Conservation, Department of Biology, Plant Conservation and Population Biology, KU Leuven, Leuven, Belgium; Universitat Leipzig, GERMANY

## Abstract

Currently, one of the most important challenges is to provide sufficient and affordable food and energy for a fast-growing world population, alongside preserving natural habitats and maintaining biodiversity. About 35% of the global food production depends on animals for pollination. In recent years, an alarming worldwide decline in pollinators has been reported, putting our food production under additional pressure. Therefore, there is an urgent need to find sustainable ways to ensure this crucial ecosystem service. Recent studies have shown that floral nectar is generally colonized by microorganisms, specifically yeasts and bacteria, which may alter nectar chemistry and enhance attraction of pollinators. In this study, we investigated changes in pollinator foraging behavior and pollination success in European pear (*Pyrus communis* L.) cultivars ‘Regal Red’ and ‘Sweet Sensation’ (red sports of ‘Doyenné de Comice’) after flower inoculation with the typical nectar-inhabiting microorganisms *Metschnikowia reukaufii* and *Acinetobacter nectaris*, and a combination of both. Pollination success was monitored by measuring the number of flower visits, fruit set and seed set in two consecutive years, 2019 and 2020. Results revealed that application of a mixture of *M*. *reukaufii* and *A*. *nectaris* resulted in significantly higher visitation rates of honeybees and hoverflies. By contrast, no effects on flower visits were found when yeasts and bacteria were applied separately. Fruit set and seed set were not significantly affected by any of the inoculation treatments. The only factors affecting fruit set were initial number of flower clusters on the trees and the year. The absence of treatment effects can most likely be attributed to the fact that pollination was not a limiting factor for fruit set in our experiments. Altogether, our results show that inoculation of flowers with nectar microbes can modify pollinator foraging patterns, but did not lead to increased pollination success under the conditions tested.

## Introduction

One of the biggest challenges today is to ensure the production of sufficient and affordable food and energy for a fast-growing world population, alongside preserving natural habitats and biodiversity [1,2]. About 90% of the world’s flowering plants, including 75% of all crops, and about 35% of the world’s food depend on animal pollinators to reproduce [3,4]. In recent years, an alarming worldwide decline in wild pollinators has been reported, probably because of changes in land use and the application of pesticides and other pollutants [1,3]. This translates into farmers increasingly relying on pollination by introduced pollinators such as honeybees, mason bees and bumblebees. Nevertheless, despite the introduction of commercial pollinators, insufficient or inefficient pollination is still a common cause of low yields in several plant species [5–8]. Therefore, there is an urgent need for alternative ways to enhance and ensure this crucial ecosystem service.

Plants use different strategies to attract pollinators, including flower color, size, shape and scent, and they often reward visiting insects with nectar and pollen [9]. Floral nectar mainly consists of sugars, especially sucrose, glucose and fructose, thereby representing an important source of food for most animal pollinators. In many cases, nectar sugar composition is an indicator for the type of pollinator. For example, sucrose-rich nectar has often been recorded in flowers pollinated by hummingbirds and insects with long mouthparts (e.g. long-tongued bees and butterflies), whereas hexose-rich nectars commonly occur in flowers pollinated by short-tongued bees, flies and bats [10–12]. In addition to sugars, floral nectar contains small amounts of amino acids, lipids, secondary metabolites and volatile organic compounds, which may influence nectar attractiveness [10,13]. Apart from the chemical composition, also nectar physical characteristics mediate flower attraction. For example, honeybees prefer warmer nectar because it has a lower viscosity which is easier to drink, and also reduces thermoregulatory costs [14,15].

Given its high sugar content, nectar also provides an ideal habitat for microbes. In fact, although nectar is assumed to be initially sterile following flower opening [16], it very quickly becomes colonized by microorganisms, particularly yeasts and bacteria, vectored by visiting pollinators or wind. These microbes can change nectar chemical and physical characteristics, and thus affect nectar attractiveness and pollination success [17–19]. The presence of microorganisms in floral nectar has been shown to decrease sugar concentrations, alter sugar composition, influence acidity, reduce concentrations of secondary metabolites, and change amino acid content and concentration [20]. Yeasts inhabiting floral nectar have also been shown to increase the temperature within flowers by the metabolic heat they produce [21]. Additionally, nectar-inhabiting yeasts and bacteria have the potential to change nectar odor by the production of volatile organic compounds [22–24], making the flowers more attractive for certain pollinators [18,25,26]. An increase in foraging activity of pollinators has been observed when yeasts were present in nectar of the late winter-early spring flowering *Helleborus foetidus* and different larkspurs [27–29]. Especially bumblebees have been shown to forage longer on flowers whose nectar is colonized by yeasts, which may enhance pollination [29]. Nevertheless, there is also some evidence that certain pollinators are more likely to avoid nectar colonized by bacteria [17,30,31]. In these studies, however, effects were tested for yeasts and bacteria separately, while their potential interaction effects have been underexplored so far [32,33].

Because nectar-inhabiting microbes have the potential to improve plant pollination by altering nectar properties, nectar manipulation through inoculation of microbes shows great potential to increase pollination and crop yield. This is especially the case in crops that produce nectar with low sugar concentrations, which decreases attractiveness to pollinators, such as pear [34,35]. Most pear cultivars are self-incompatible and depend on insect pollination for sufficient fruit set. Compared to other fruit crops such as apple and cherry, pear flowers usually produce small amounts of nectar [35]. Pollinators like honey bees visiting pear blossoms therefore often switch to other, more rewarding plants, resulting in insufficient pollination and poor pear yields. Moreover, seed formation, which increases with insect pollination, affects fruit quality. Pears with large numbers of seed are known to have a higher fruit mass and a more optimal shape and flavor than those with low numbers of seeds [36,37].

In this study, we aimed to investigate changes in pollination behavior in European pear (*Pyrus communis* L.) cultivars ‘Regal Red’ and ‘Sweet Sensation’ (red sports of ‘Doyenné de Comice) after inoculation of typical nectar microbes, including *Metschnikowia reukaufii* and *Acinetobacter nectaris*. Specifically, newly opened pear flowers were inoculated with each microbial species separately, or a combination of both species by hand spraying the inoculum into the flowers. Subsequently, flower visits by bees and hoverflies were monitored during flowering, and pollination success was measured by quantifying fruit and seed set. The experiment was performed in two consecutive years, 2019 and 2020.

## Material and methods

### Microbial species and inoculum preparation

Two common nectar-inhabiting microbial species were used in this study, *Metschnikowia reukaufii* (Ascomycota, Metschnikowiaceae) and *Acinetobacter nectaris* (Proteobacteria, Moraxellaceae). *Metschnikowia reukaufii* is one of the most common yeast species in floral nectar [20,38] and has also been retrieved from bees, ants, hummingbirds and other pollinators [39–41]. Behavioral tests under both controlled greenhouse conditions and field conditions have shown that bumblebees prefer nectar inoculated with *M*. *reukaufii* to non-inoculated nectar [19,27,29]. Furthermore, recent work has shown that *M*. *reukaufii* produces distinct volatile organic compounds that are attractive to flower-visiting insects such as bees [18,26] and parasitic wasps [42,43]. *Acinetobacter nectaris* also commonly occurs in floral nectar [44–47], often in combination with *M*. *reukaufii* [48]. The strains used in this study, *M*. *reukaufii* CdV Mar 33.1 [49] and *A*. *nectaris* LMG 26958, were isolated from floral nectar of *Gladiolus italicus—communis* (Iridaceae; collected in Morocco) and *Muscari comosum* (Hyacinthaceae; Spain), respectively. Both strains were stored in 37.5% glycerol at −80°C.

In order to prepare the inocula, strains were first plated on yeast extract peptone dextrose agar (YPDA) (*M*. *reukaufii*) and trypticase soy agar (TSA) (both from Oxoid, Basingstoke, UK) supplemented with 5% fructose (*A*. *nectaris*). Next, plates were incubated at 25°C and a subculture was made on the same medium after two days. Subsequently, following an incubation period of four days at 25°C, three 250 ml-Erlenmeyers filled with 150 ml yeast extract peptone dextrose (YPD; Oxiod) and 150 ml trypticase soy broth (TSB; Oxoid) were inoculated with the yeast and bacterial strain, respectively. After an incubation period of three days at 25°C on a shaking platform (120 rpm), cells were transferred into fresh growth medium. Specifically, for each strain 50 ml of homogenized culture medium was added to eight 1 l-Erlenmeyers with 750 ml YPD (*M*. *reukaufii*) or 750 ml TSB (*A*. *nectaris*), and again incubated under the same conditions. After five days cell suspensions were centrifuged for 9 min at 3,500 g. Pellets were then combined and suspended in tap water to a total volume of 7 l yeast suspension and 7 l bacterial suspension, and immediately used for flower inoculation.

### Study sites and inoculation of pear blossoms

The field studies in this research were performed in pear orchards with consent of the owners. Experiments were performed in Belgium in 2019 and 2020 in three commercial pear orchards, including two orchards with cultivar ‘Sweet Sensation’ and one orchard with cultivar ‘Regal Red Comice’. Both cultivars are red sports of the self-incompatible, late-flowering ‘Comice’ pear cultivar with a short effective pollination period of a few days depending on temperature and flower quality [36,50]. In 2019, the experiment was performed in Tielt-Winge (50°54’ N 4°56’ E), Gutshoven (50°46’ N 5°19’ E) (both ‘Sweet Sensation’), and Herk-de-Stad (50°55’ N 5°09’ E) (‘Regal Red Comice’). In 2020, the experiment was conducted in the same orchards except from the one in Gutshoven, which was replaced by a ‘Sweet Sensation’ orchard in Horpmaal (50°45’ N 5°19’ E). In the pear orchards in Gutshoven and Herk-de-Stad pollinizer trees of cultivars ‘Conference’ (fully-compatible) and ‘Concorde’ (semi-compatible), respectively, were present at a density of one tree per ten trees. In the orchards in Tielt-Winge and Horpmaal pollinizer trees of cultivar ‘Conference’ were present in separate rows (1 on 4 rows and 1 on 5 rows, respectively). In all orchards two honeybee hives and ten nesting blocks for mason bees each containing *c*. 200 bamboo tubes as nesting material were provided per hectare to support pollination. The honeybee hives were put in the orchards at the beginning of pear flowering in each study year. The nesting blocks were already introduced in 2017 in order to have adequate population sizes of mason bees when performing the experiments. In the orchard in Horpmaal, the nesting block was put in the field in 2018. At the beginning of the experiment the occupancy rate (number of occupied bamboo sticks/total number of bamboo sticks) of the nesting blocks was between 40 and 50% (containing 600–700 female mason bees per ha based on a test sample). In each orchard, four treatments were applied. These included a treatment in which yeast cells were applied, a treatment with bacterial cells, and a combination of both. A treatment in which tap water was applied was included as a control. Each treatment was repeated in each orchard in four plots of five pear trees per plot. Plots were separated by a distance of at least 15 m along the row and 10 m across rows (S1 Fig). In Gutshoven and Herk-de-Stad, each plot also contained one pollinizer tree in the middle of the plot. In Tielt-Winge and Horpmaal, the plots were constructed in rows next to a pollinizer row (4 m between rows). Treatments were applied on a sunny dry day when 90% of the pear blossoms were flowering, i.e. on the 11^th^ of April 2019 and the 9^th^ of April 2020. To this end, in the morning of the day of inoculation, a total of 75 ml cell suspension was sprayed using a hand sprayer on all flowers of the tree (similar average amount of flowers per tree between treatments: *c*. 660 in 2019 and 840 in 2020). For the combined treatment of yeasts and bacteria, a combination of 37.5 ml of each suspension was applied. In 2019 the OD_600_ values for the yeast and the bacterial suspensions were 0.7 and 0.35 (corresponding with 1.1 x 10^7^ and 4.2 x 10^7^ CFU (colony forming units)/ml, respectively; in 2020 they were 0.9 and 0.5 (corresponding with 1.5 x 10^7^ and 7.1 x 10^7^ CFU/ml), respectively.

### Transect walks

In order to assess potential differences in insect attraction among treatments, transect walks were carried out in each orchard at five time points between 10 am and 5 pm in the first three days after microbial inoculation. At each time point, each plot of five trees was observed for 2 min at the sunny side of the trees, and only effective flower visits were noted, i.e. when the anthers or stigmas were touched by an insect. In each orchard, for each treatment and each time point, the sum of effective flower visits of the four plots was then made, representing the number of effective flower visits in a total of 8 min for each transect walk. This value was recalculated to visits per tree per hour (dividing by 20 and multiplying by 60/8). A distinction between taxonomic groups of pollinators was made as follows: honeybees, bumblebees, horned mason bees, other bees, and hoverflies.

### Fruit set and seed set

In March 2019 and 2020, the initial number of flower clusters on the 20 trees per treatment per orchard was counted to estimate the initial number of flowers, i.e. 7.5 times the number of clusters per tree (based on flower counts of previous years). Fruit set, i.e. the percentage of flowers developing into mature fruits, was monitored on individual trees after the June drop for all 20 trees per treatment per orchard. The June drop refers to the natural tendency of fruit trees to drop immature fruit after flowering, usually around the beginning of June, to avoid over-bearing of the tree. In 2019 and 2020 fruits were harvested on the 2^nd^ of September and the 23^rd^ of August, respectively. Two fruits per tree (40 fruits per treatment) in each orchard were randomly sampled to quantify seed set. Seed set was defined as the number of well-developed seeds present in the fruit.

### Confirming presence of inoculated microorganisms

To confirm the presence of the applied microbes after inoculation of the flowers, in each orchard six samples of ten random flowers each were collected for every treatment 30 h after inoculation and analyzed by qPCR. For each sample, genomic DNA was extracted from 0.5 g homogenized flower material using QIAGEN DNeasy PowerSoil Kit (Qiagen, Hilden, Germany) according to the manufacturer’s instructions with one modification: in the third and fourth step of the protocol the use of a vortex adapter was replaced by two cycles of 30 s in the Precellys 24 homogenizer (Bertin instruments, Montigny-le-Bretonneux, France) at 4000 rpm. Subsequently, DNA was subjected to a qPCR analysis to quantify *M*. *reukaufii* and *A*. *nectaris* DNA in the samples. Reactions were carried out in an ABI StepOnePlus real-time PCR system (Applied Biosystems, Carlsbad, CA, USA) in a total volume of 20 μl containing 1 μl DNA, 0.2 μl of each primer (20 μM stock), 10 μl 1x SsoAdvanced Universal SYBR Green Supermix and 8.6 μl nuclease free water. Amplification was performed using the primers Mr_LSU_F1_2 (5’-TGC AAG CAG ACA CAA CCT CG-3’) and Mr_LSU_R1_2 (5’-CGC CAG CAT CCT TGA AGA AT-3’) and An_rpoB_F1_1 (5’-AAA CGT GGT GAT CAG CTG ACT-3’) and An_rpoB_R1_1 (5’-GTA AGA CGT TCA GCG ATA C-3’), targeting the large subunit of the ribosomal RNA (rRNA) gene and the gene encoding the RNA polymerase subunit B (*rpoB*) of *M*. *reukaufii* and *A*. *nectaris*, respectively. Thermal cycling conditions consisted of an initial denaturation step at 95°C for 10 min, followed by 40 cycles of a denaturation step at 95°C for 15 s, an annealing at 57°C for 60 s and an elongation step at 72°C for 30 s. Fluorescence (520 nm) was detected at the end of the elongation phase for each cycle. The threshold cycle (C_T_), or the PCR cycle where fluorescence was first detected, was determined automatically using the Applied Biosystems software. The baseline was set automatically, while the threshold was manually set at 0.52 (which was above any background). At the end of each run, a melting curve analysis was performed to confirm amplification specificity. In each analysis, a negative control (DNA replaced by DNA-free water), and a standard ten-fold dilution series of genomic DNA of *M*. *reukaufii* CdV Mar 33.1 and *A*. *nectaris* LMG 26958 was included. All reactions were performed in duplicate. The amount of target DNA in each sample was calculated from the standard curve and expressed as ng target DNA per gram flower material.

### Statistical analysis

All statistical analyses were performed in R version 4.0.2 (R Development Core Team, 2020). The effects of the different inoculation treatments on pollinator visits, fruit set and seed set were analyzed using generalized linear mixed models (GLMMs) with a Gauss, beta and negative binomial distribution, respectively, using the glmmTMB package [51]. Inoculation treatment was included as fixed factor and orchard as random factor. The initial number of flower clusters on the tree was added as covariate when modelling fruit set. When modeling flower visit data, time (5 time points) was added as random factor, and when modelling fruit and seed set data, plot number was added as random factor. When using a beta distribution, 0 and 1 are not allowed as data points. Therefore the fruit set data (Y) were transformed as follows: Y’ = (Y*(N-1)+0.5)/N, in which N stands for the sample size [52]. The most parsimonious model for fruit set was selected using Akaike’s information criteria. When the inoculation treatment had a significant effect, a post hoc Tukey test was performed using the glht function of the multcomp package [53] in order to perform pairwise comparisons.

The effect of inoculation treatment (fixed factor) on the presence of *M*. *reukaufii* and *A*. *nectaris* was studied using GLMM’s with a Gauss distribution (glmmTMB package [51]), in which orchard was included as random factor. Again a post hoc Tukey test was carried out using the glht function of the multcomp package [53] to perform pairwise comparisons.

## Results

### Transect walks

**Fig 1:**
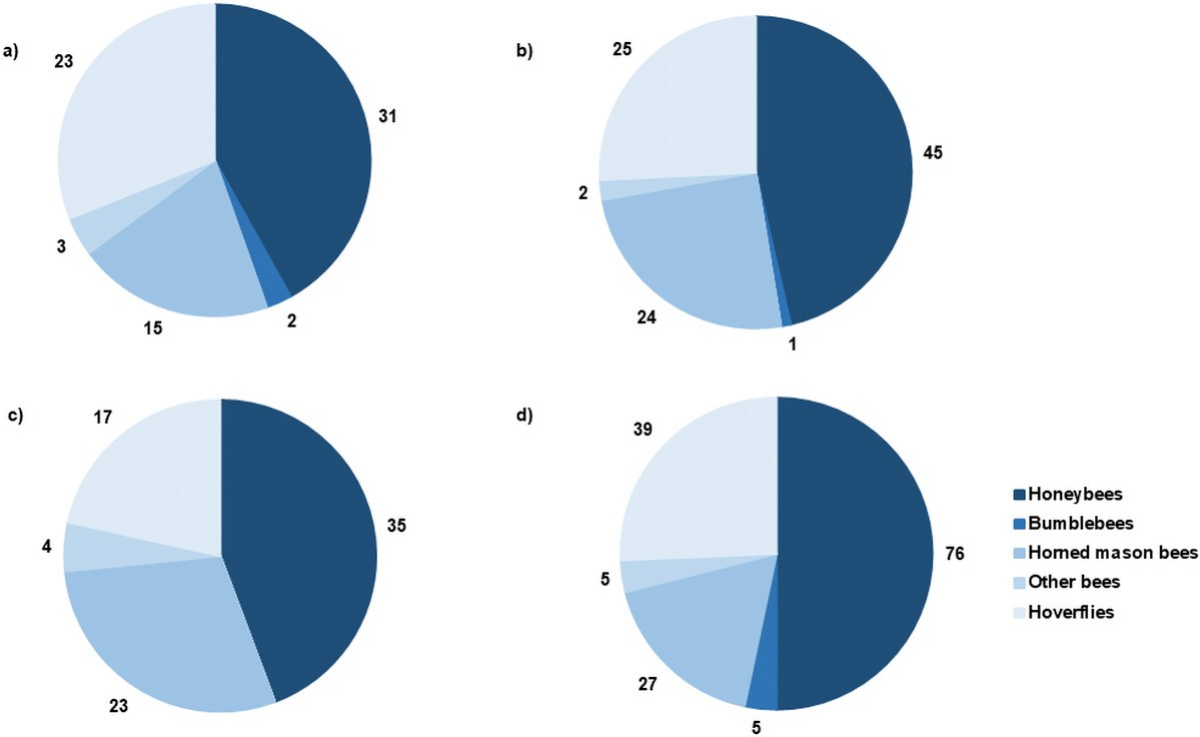
Distribution and total number of insects monitored during transect walks at five time points following microbial inoculation of flowers (data from 2019 and 2020 combined). Treatments included inoculation of flowers with the yeast *Metschnikowia reukaufii* (b), the bacterium *Acinetobacter nectaris* (c), and a combination of both (yeast + bacteria (d)). As a control, flowers were sprayed with water (a).

Honeybees were the most abundant flower visitors recorded across the different treatments, followed by hoverflies and mason bees (Fig 1). In 2019 no bumblebees were observed visiting pear flowers during transect walks, and in 2020 only eight individuals were observed, five on trees with the yeast + bacteria treatment, two on trees with the control treatment and one on trees with the yeast treatment. When zooming in at the different treatments, most insects (for all five categories) were observed for the yeast + bacteria treatment (Fig 1d).

**Fig 2:**
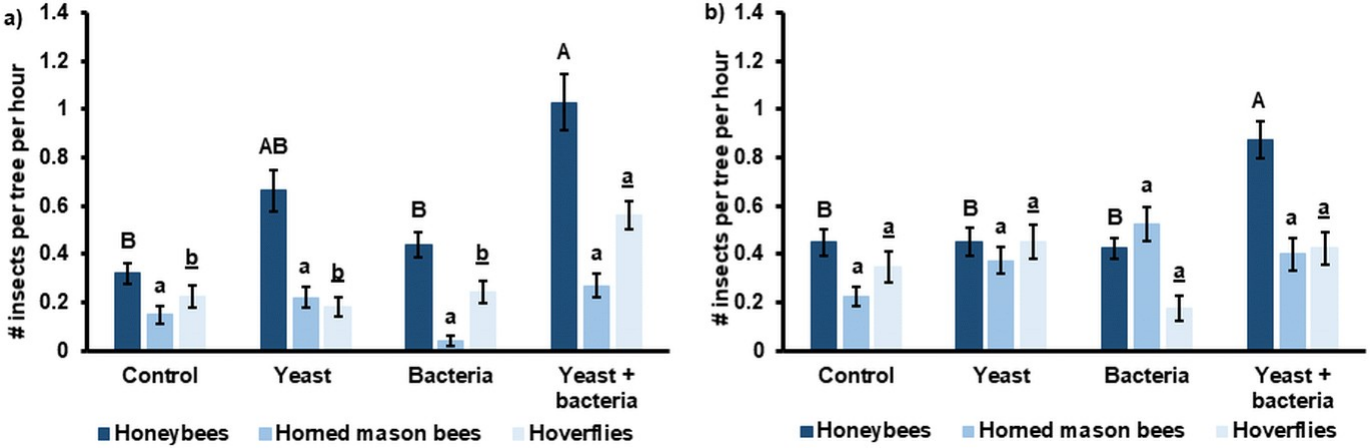
Average number of honeybees, horned mason bees and hoverflies foraging per tree per hour (N = 15) for each inoculation treatment applied in 2019 (a) and 2020 (b). Treatments included inoculation of flowers with *Metschnikowia reukaufii* (yeast), *Acinetobacter nectaris* (bacteria), and a combination of both (yeast + bacteria). As a control, flowers were sprayed with water. Statistics were performed separately on each pollinator group, indicated with different letter fonts: capitals for honeybees, lower case for horned mason bees and lower case underlined for hoverflies. Treatments with different letters within one pollinator group differ significantly (P < 0.05). Error bars represent standard errors of the mean.

In both years, honeybees were significantly more observed in the yeast + bacteria treatment compared to the control (2019: P < 0.001; 2020: P = 0.047) and the bacteria treatment (2019: P = 0.008; 2020: P = 0.030) (Fig 2). Only in 2020 there was also a significant difference between the yeast + bacteria and the yeast treatment (P = 0.046). In 2019, also hoverflies were significantly more abundant in the yeast + bacteria treatment compared to the control (P = 0.044), yeast (P = 0.016) and bacteria treatment (P = 0.05) (Fig 2). Only a few horned mason bees, especially in 2019, were seen on the trees in both years, with no significant differences between treatments (Fig 2).

### Fruit set and seed set

**Fig 3:**
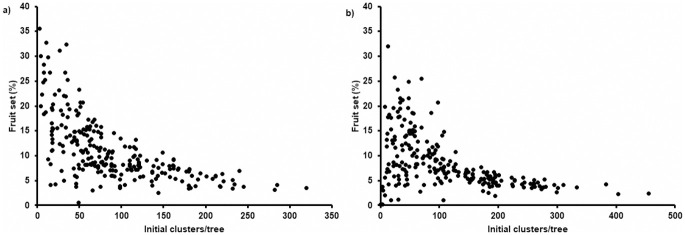
Fruit set (%, (number of fruitlets after June drop/number of flowers)*100) per tree in function of the initial number of flower clusters on the trees recorded in 2019 (a) and 2020 (b).

**Fig 4:**
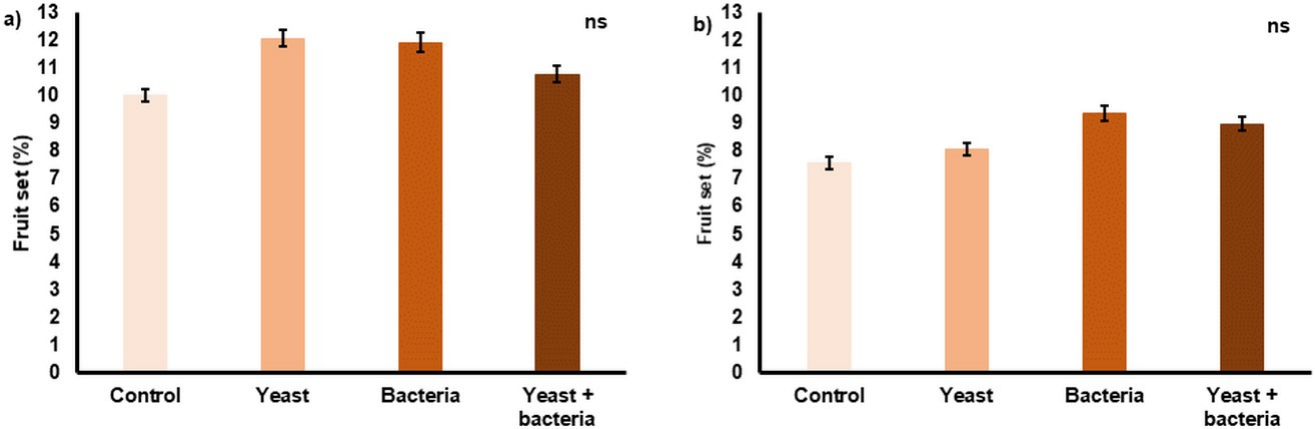
Average fruit set (%) per tree (N = 60) for each inoculation treatment applied in 2019 (a) and 2020 (b). Treatments included inoculation of flowers with *Metschnikowia reukaufii* (yeast), *Acinetobacter nectaris* (bacteria), and a combination of both (yeast + bacteria). As a control, flowers were sprayed with water. There were no significant (P > 0.05) differences among treatments (ns). Error bars represent standard errors of the mean.

**Fig 5:**
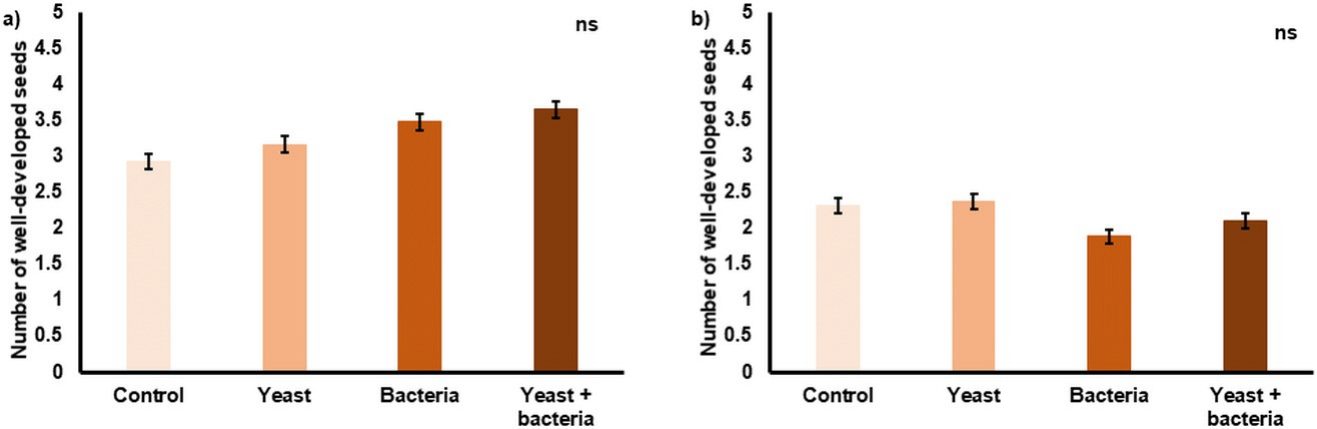
Average seed set per fruit (N = 120) for each inoculation treatment applied in 2019 (a) and 2020 (b). Treatments included inoculation of flowers with *Metschnikowia reukaufii* (yeast), *Acinetobacter nectaris* (bacteria), and a combination of both (yeast + bacteria). As a control, flowers were sprayed with water. There were no significant (P > 0.05) differences among treatments. Error bars represent standard errors of the mean.

Fruit set was significantly higher (P < 0.0001) in 2019 (11.2 ± 6.4%) compared to 2020 (8.5 ± 5.4%) and was significantly affected by the initial number of flower clusters per tree (P < 0.0001): a lower number of initial clusters resulted in a higher fruit set (Fig 3). Both in 2019 and in 2020 no significant differences among treatments were found for fruit set (Fig 4) and seed set (Fig 5).

### Yeast and bacterium presence after inoculation

**Fig 6:**
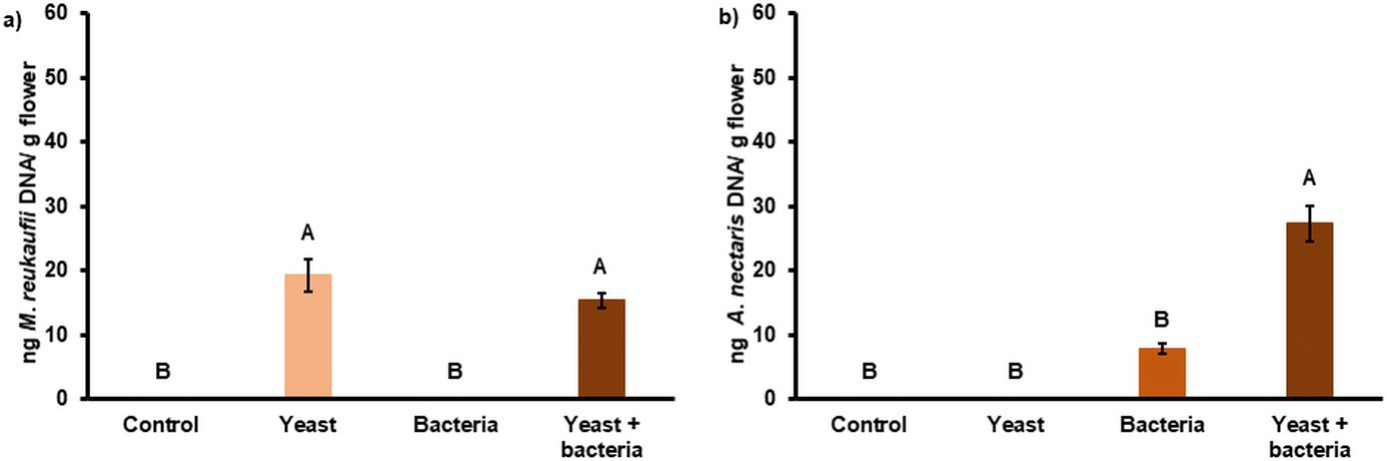
Average DNA amount (N = 18) of *Metschnikowia reukaufii* (a) and *Acinetobacter nectaris* (b) in pear flowers one day after inoculation. Treatments included inoculation of flowers with *M*. *reukaufii* (yeast), *A*. *nectaris* (bacteria), and a combination of both (yeast + bacteria). As a control, flowers were sprayed with water. Treatments with different letters differ significantly (P < 0.05). Error bars represent standard errors of the mean.

qPCR analysis confirmed the presence of the inoculated microbes in the sprayed flowers and absence in the control flowers (Fig 6). Although *A*. *nectaris* was inoculated at half of the dose in the yeast + bacteria treatment, the bacterium was significantly more abundant on flowers of the yeast + bacteria treatment compared to the flowers where only the bacteria were inoculated (P = 0.003) (Fig 6).

## Discussion

In this study, we investigated changes in pollinator foraging behavior and pollination success in European pear after flower inoculation with the nectar microorganisms *M*. *reukaufii* and/or *A*. *nectaris*. Previous experiments under both controlled greenhouse conditions and field conditions have shown that bees prefer nectar inoculated with *M*. *reukaufii* over non-inoculated nectar [19,27,29], most probably because they can find this nectar more easily due to a more complex olfactory display [18] or they find this nectar more rewarding [21]. Our results revealed that application of a mixture of *M*. *reukaufii* and *A*. *nectaris* resulted in higher insect visitation rates, although differences were only significant for honeybees (in both 2019 and 2020) and hoverflies (2019). In contrast, no effects were seen when yeasts and bacteria were applied separately. So far, the mechanisms underlying these synergistic effects remain unclear, but it is reasonable to assume that a more attractive mixture of volatile compounds was obtained when combining both microbes [32,54]. However, despite the fact that the volatile emission of a co-culture can be modeled in theory by summing the volatile emissions of the monocultures, a co-culture of an attractive yeast and bacterium does not always result in a more attractive volatile profile [55]. *Metschnikowia reukaufii* and *Acinetobacter* species, including *A*. *nectaris*, are commonly found together in floral nectar [48]. A potential explanation may be found in the fact that both microorganisms show complementary nutrient utilization patterns [32]. Whereas *A*. *nectaris* prefers fructose and does not grow well on sucrose and glucose, *Metschnikowia* is able to hydrolyze sucrose and consume the resulting glucose, enriching the nectar in fructose [32,45]. Likewise, in a recent study it has been found that nectar acinetobacters consume a wide diversity of nitrogen sources, including by-products of yeast metabolism [56]. Such interactions may also explain why *A*. *nectaris* was abundantly present in the yeast + bacteria treatment, while it was less abundant when only the bacteria were applied, even though at a double dose was used. While several studies have focused on the consequences of either nectar yeasts or bacteria for pollination [17,27,28,31], so far only very little is known about the effects of yeast-bacterium interactions. Nevertheless, recent evidence suggests that such interactions are important drivers of the assembly of nectar microbial communities and may have important consequences for pollination [32,33]. Although our results suggest that a combination of yeasts and bacteria may outperform an application of yeasts or bacteria separately, further research is needed to investigate how and to which extent yeast-bacterium interactions affect pollination and plant reproduction.

Although highest insect visitation rates were recorded for the combined treatment of *M*. *reukaufii* and *A*. *nectaris*, the total number of insect visits was still low (on average 2.0 insect visits per hour per tree) compared to what is generally considered sufficient for adequate pollination. The recommendations by Mayer et al. [57] for adequate pollination of pear include 10–15 honeybee flower visits per tree per minute. However, it should be noted that these numbers are representative when pollination is performed by honeybees only, whereas other managed bees and wild bees may also contribute to pollination [36]. Moreover mason bees and bumblebees are known to be more efficient (more effective stigma contacts) pollinators than honeybees, especially nectar-foragers [36,58], which may reduce the total number of flower visits needed for adequate pollination. In their study on different pear cultivars, including ‘Comice’, in which they introduced honeybees and bumblebees in the orchard, Quinet et al. [35] reported that a total of five insect visits per tree per minute (of which 50% were honeybees, 15% hoverflies, 15% bumblebees and 20% other bees) resulted in a valuable fruit- and seed set. In our study, on average only one honeybee per tree per hour was monitored on the trees treated with *M*. *reukaufii* and *A*. *nectaris*. The maximum number of honeybees monitored in a single plot was eight per 8 min, which equals 12 honeybees per tree per hour. On that day the temperature reached 16°C. On time points when the temperature only reached 10°C no honeybees were seen. Honeybees are known to only start foraging at temperatures of 12–13°C [59], which may have had an effect on our results. We should remark that, although timed transect walks are generally used to estimate insect abundance [60], they only provide snapshot data and may not be a good representation of the flower visits during the whole flowering period. Measurements that are more accurate may be obtained by camera observations that give a more accurate account of the flower visits that occurred over a longer period.

In 2019 hoverflies were also more abundantly present on trees with the yeast + bacteria treatment. Hoverflies are important pollinators of wild flowers but also visit several crops [61]. Several studies [7,35,62] reported hoverflies visiting flowers and making contact with their reproductive parts. Hairier hoverflies are known to carry 3500–10000 pollen grains [63] but the contribution of hoverflies to pear pollination is not well studied. Microbial inoculation did not have a significant effect on mason bee flower visits. Overall, despite the presence of mason bee nesting blocks the number of mason bees observed on the pear trees was low (< 1 mason bee/tree/hour), especially in 2019. In their study on mason bee foraging behavior on ‘Comice’, Monzon et al. [36] reported 75 flower visits on 32 trees over a period of 40 min per tree, corresponding to 3.5 mason bee visits per tree per hour. It is not clear why mason bee counts were low in our study. Most likely this can be explained by the early emergence of the mason bees (three weeks before flowering) and the lack of other food resources close to the orchard at that moment [64].

Despite the higher number of insect visits, inoculation of pear flowers with a combination of *M*. *reukaufii* and *A*. *nectaris* did not result in an increased fruit set or seed set, nor did the other treatments. The only studied factors significantly influencing fruit set were year and initial number of flower clusters. In 2019, fruit set was overall significantly higher compared to 2020. This can be explained by the fact that circumstances for setting fruit were better in 2019. Indeed, in 2020 there was a severe cold period in the night of March 31^st^. At that point clusters were in the white bud stage having a critical temperature of about -3°C, below which crucial flower damage occurs [65]. During that night, temperature dropped to -4°C in Tielt-Winge, -4.7°C in Horpmaal and -2.5°C in Herk-de-Stad. Not only temperature but also duration of exposure to low temperatures play a role in the damage that occurs [66]. In Horpmaal, temperature remained below -3°C for over 4 h compared to 2 h in Tielt-Winge and 0 h in Herk-de-Stad. This is reflected in the percentage of damaged flowers in the studied orchards. In Horpmaal, around 70% of the flowers in the lower part of the tree were damaged within the ovules, while this was only around 25% for the orchards in Herk-de-Stad and Tielt-Winge (S1 Dataset). Consequently, this led to less flowers in 2020 that could develop into mature pears. Furthermore, seed set data suggest that there was more pollination in 2019 compared to 2020, although differences were not significant. In 2019, pears contained an average of 3.3 seeds compared to 2.2 seeds in 2020. When looking into flower visit data, slightly more honeybees were seen in 2019 but less mason bees. In addition to year, which is most likely reflected in different weather conditions, initial number of flower clusters significantly affected fruit set. When there are more flower clusters on a tree, more competition between developing fruitlets and other growing plant organs such as shoots and leaves occurs, resulting in a higher percentage of fruit drop, and hence a relatively lower fruit set [36,67]. This was also observed in our study and can be illustrated using the 2020 data of the orchards in Horpmaal and Tielt-Winge. In Horpmaal the initial number of flowers was three times higher than in Tielt-Winge, on average 1500 flowers (200 clusters) compared to 500 flowers (63 clusters) (S2 Fig). Hence the fruit set in Horpmaal was lower (5%) compared to Tielt-Winge (8%), while the production in Horpmaal was higher (64 fruits compared to 32 in Tielt-Winge). In general, an average of 30 fruits per 100 flower clusters is considered a good fruit set when the initial amount of clusters is high [68,69]. In our study, values varied between 28 and 58 pears per 100 flower clusters. Therefore, getting significant differences in fruit set between the different treatments was most probably unlikely, as all trees were at their maximum production limit controlled by fruit drop. Moreover, fruit set obtained after hand pollination in Tielt-Winge (9.9 ± 3.4%) (S2 Dataset) was not significantly higher compared to the fruit set obtained in our experiment for the control treatment (no inoculation) (7.2 ± 3.3%), reinforcing that the trees were at maximum production capacity. This was also observed in previous studies in ‘Doyenné du Comice’ indicating that pollination is not always the limiting factor for fruit set in this cultivar [70,71]. Therefore, the problems regarding fruit set that arise in this cultivar may be due to genetic, nutritional and/or other factors.

In general, seed set was relatively high in our study, especially in 2019, with average values between 2.9 and 3.6 seeds per pear. A seed set of two to three seeds per fruit is generally considered a good indicator for high quality ‘Comice’ pears, having a better quality compared to fruits without developed seeds [7,36]. Microbial inoculation did not affect seed set. Seed set highly fluctuated between fruits from zero to nine seeds per fruit, with the same pattern for all inoculation treatments, resulting in large standard variations (S3 Fig).

The limited research on the effect of *M*. *reukaufii* on plant reproductive success showed different results in different plants, but no positive effects on fruit and seed set were reported so far. Herrera et al. [27] reported a lower fruit and seed set of *H*. *foetidus* after inoculation of the nectar with *M*. *reukauffi* due to longer pollinator visits resulting in an increase of self-pollen. On the other hand, Vannette et al. [30] and Schaeffer and Irwin [28] found no detrimental effects of *M*. *reukauffi* on fruit-and seed set in *Mimulus aurantiacus* and *Delphinium nuttallianum* flowers respectively.

Altogether, we have shown that inoculation of pear flowers with a combination of the nectar microbes *M*. *reukaufii* and *A*. *nectaris* resulted in a higher visitation rate of honeybees and hoverflies, while this was not the case when the microbes were inoculated separately. Nevertheless, despite increased insect visitation rates, fruit set and seed set were not increased, most probably because trees were at maximum production capacity. We expect that under poor pollination conditions (e.g. cold, rainy days) and when trees have a limited number of flowers, microbial inoculation may lead to more pronounced effects. Further research is needed to confirm this. Then, nectar inoculation with microbes may be an effective way to enhance pollination and crop yield, which will be especially beneficial for crops that produce nectar with low sugar concentrations like pear.

## Supporting information

S1 Fig Experimental set-up. Experiments were performed in a pear orchard in Tielt-Winge (a), Herk-de-Stad (b), Horpmaal (c) and Gutshoven (d), and included four plots of five trees that were treated. Treatments included inoculation of flowers with *Metschnikowia reukaufii* (yeast), *Acinetobacter nectaris* (bacteria), and a combination of both (yeast + bacteria). As a control, flowers were sprayed with water. Tree rows are indicated by a number and were separated at least 4 m from each other. In each orchard pollinizer trees were planted to ensure cross-pollination. Furthermore, in each orchard two honeybee hives and ten nesting blocks for mason bees per hectare were provided to support pollination.
(PDF)

S2 Fig Average amount of initial number of clusters per tree (n = 20) for each treatment in each orchard in 2019 (a) and 2020 (b). Treatments included inoculation of flowers with *Metschnikowia reukaufii* (yeast), *Acinetobacter nectaris* (bacteria), and a combination of both (yeast + bacteria). As a control, flowers were sprayed with water. No significant differences occurred between treatments within one orchard (p>0.05). Error bars represent standard errors of the mean.
(PDF)

S3 Fig Number of fruits in function of well-developed seeds for each of the inoculation treatments. Treatments included inoculation of flowers with *Metschnikowia reukaufii* (yeast), *Acinetobacter nectaris* (bacteria) and a combination of both (yeast + bacteria). As a control, flowers were sprayed with water.
(PDF)

S1 Dataset Amount of cluster showing frost damage in 2020 in each orchard. (XLSX)

S2 Dataset Initial amount of clusters and fruit set data of supplementary hand pollination experiment in the orchard of Tielt-Winge. (XLSX)

S3 Dataset All data sets supporting the results in the results section of this paper. (XLSX)
