# Supplementary figures and images for: Inoculation of pear flowers with Metschnikowia reukaufii and Acinetobacter nectaris enhances attraction of honeybees and hoverflies, but does not increase fruit and seed set
Source: PLoS One. 2021 Apr 22;16(4):e0250203. doi: 10.1371/journal.pone.0250203 (PMC8061982; doi:10.1371/journal.pone.0250203)

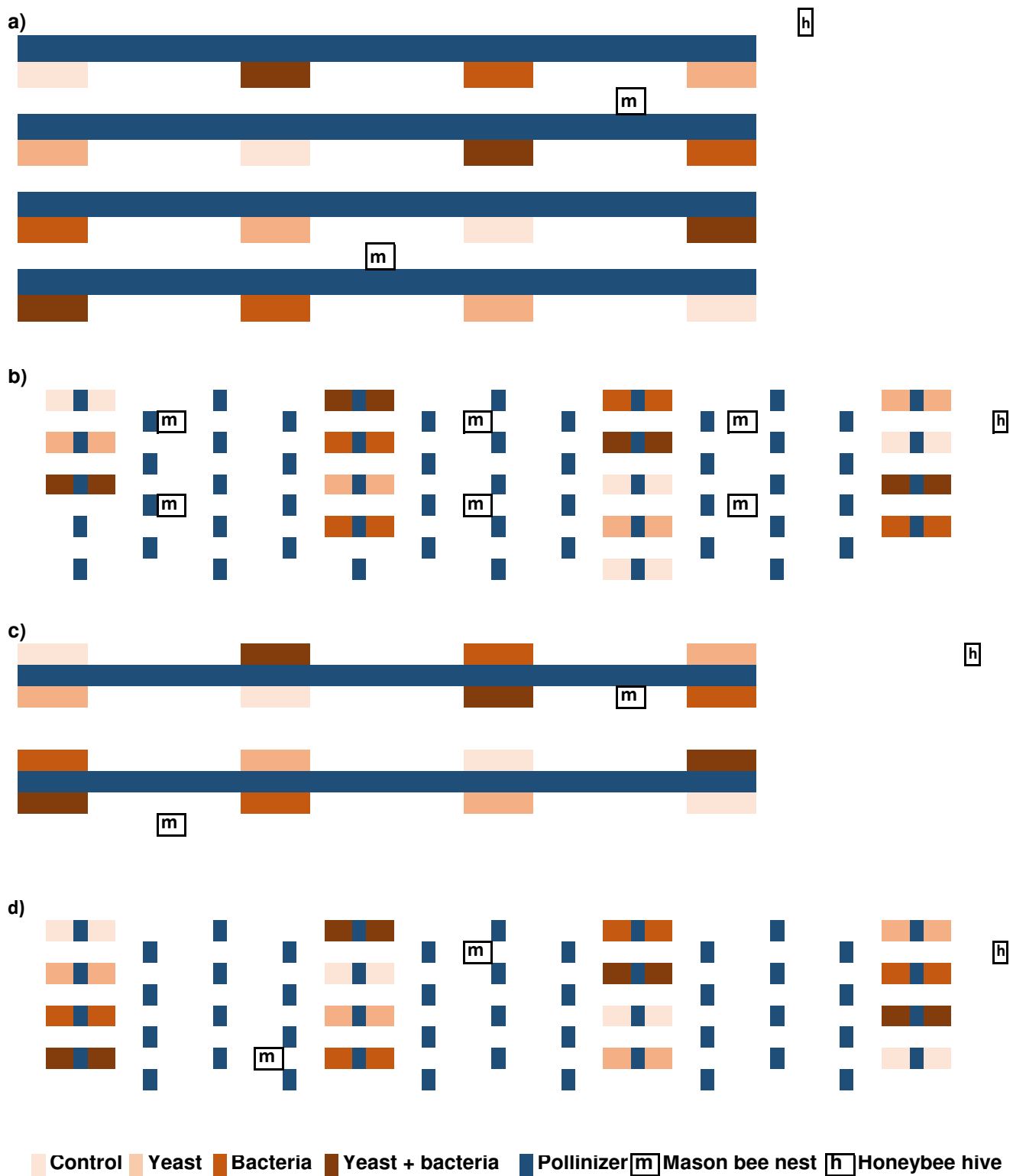

Supplement: S1 Fig — Experiments were performed in a pear orchard in Tielt-Winge (a), Herk-de-Stad (b), Horpmaal (c) and Gutshoven (d), and included four plots of five trees that were treated. Treatments included inoculation of flowers with Metschnikowia reukaufii (yeast), Acinetobacter nectaris (bacteria), and a combination of both (yeast + bacteria). As a control, flowers were sprayed with water. Tree rows are indicated by a number and were separated at least 4 m from each other. In each orchard pollinizer trees were planted to ensure cross-pollination. Furthermore, in each orchard two honeybee hives and ten nesting blocks for mason bees per hectare were provided to support pollination. (PDF) [file pone.0250203.s001.pdf]

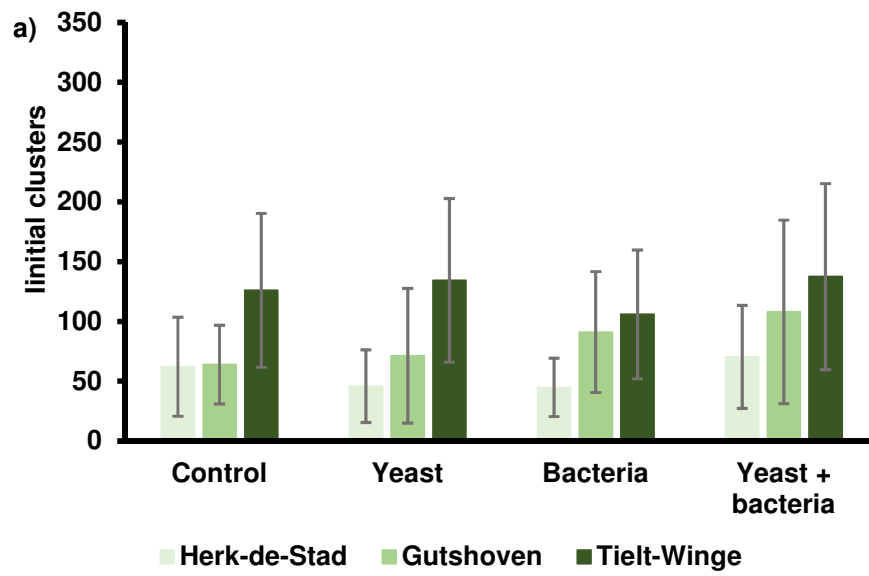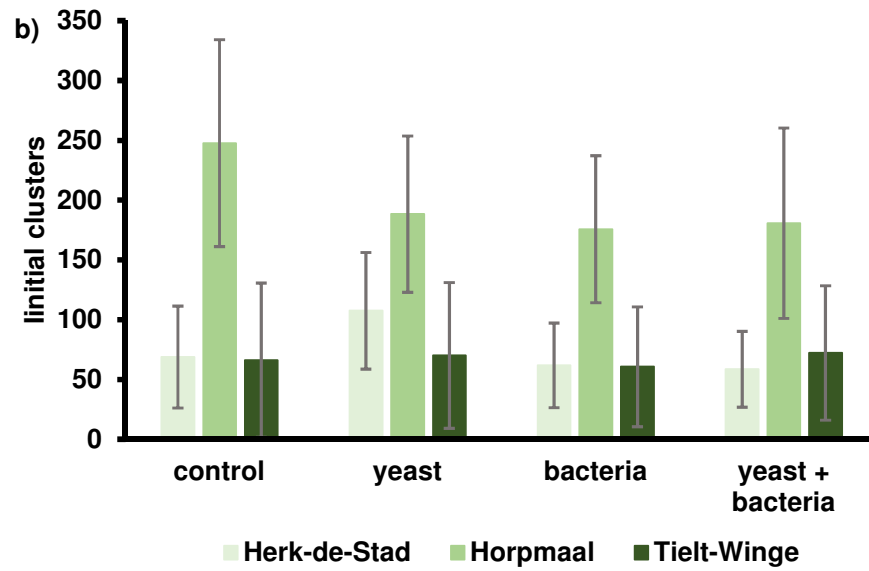

Supplement: S2 Fig — Treatments included inoculation of flowers with Metschnikowia reukaufii (yeast), Acinetobacter nectaris (bacteria), and a combination of both (yeast + bacteria). As a control, flowers were sprayed with water. No significant differences occurred between treatments within one orchard (p>0.05). Error bars represent standard errors of the mean. (PDF) [file pone.0250203.s002.pdf]

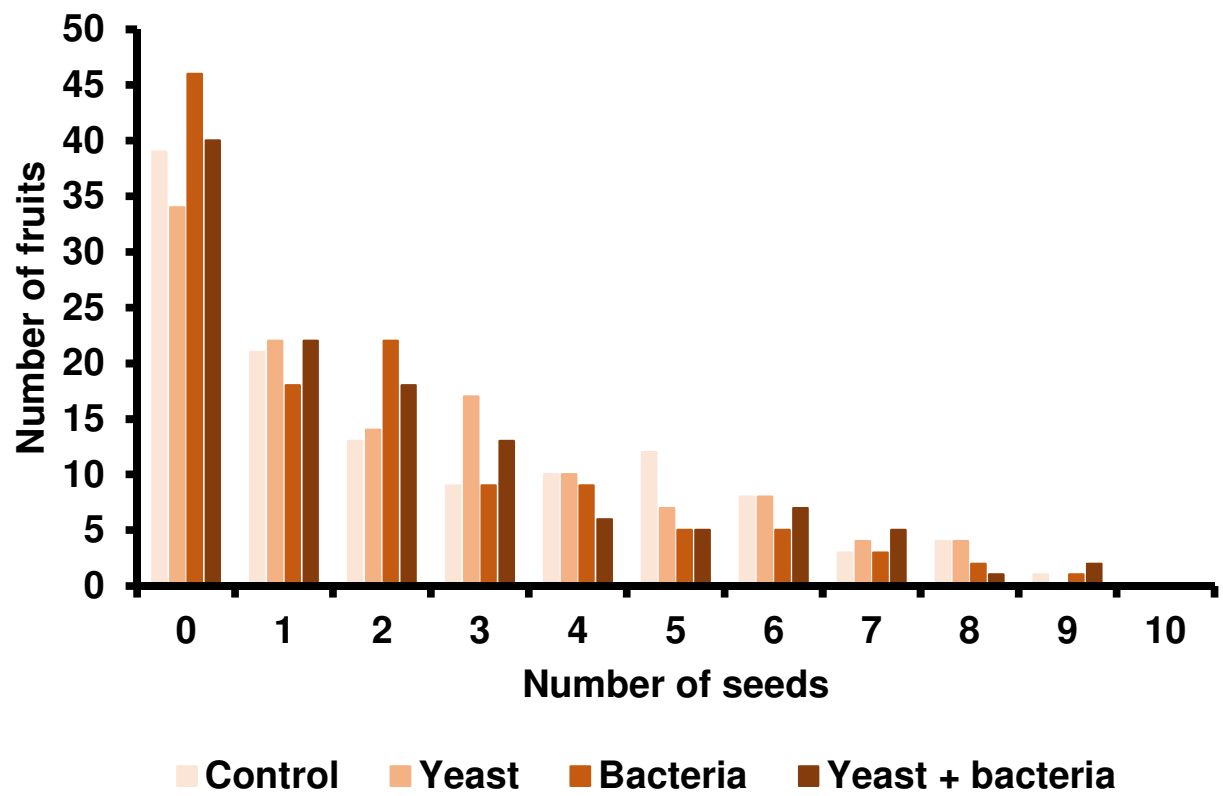

Supplement: S3 Fig — Treatments included inoculation of flowers with Metschnikowia reukaufii (yeast), Acinetobacter nectaris (bacteria) and a combination of both (yeast + bacteria). As a control, flowers were sprayed with water. (PDF) [file pone.0250203.s003.pdf]
